# Association of Medicare Mandatory Bundled Payment Reform With Joint Replacement Surgery Use for Beneficiaries With Alzheimer Disease and Related Dementias

**DOI:** 10.1001/jamahealthforum.2021.5111

**Published:** 2022-02-11

**Authors:** Caroline P. Thirukumaran, Benjamin F. Ricciardi, Xueya Cai, Robert G. Holloway, Yue Li, Laurent G. Glance

**Affiliations:** 1 Department of Orthopaedics, University of Rochester, New York; 2 Department of Public Health Sciences, University of Rochester, New York; 3 Center for Musculoskeletal Research, University of Rochester, New York; 4 Department of Biostatistics and Computational Biology, University of Rochester, New York; 5 Department of Neurology, University of Rochester, New York; 6 Department of Medicine, University of Rochester, New York; 7 Department of Anesthesiology and Perioperative Medicine, University of Rochester, New York; 8 RAND Health, RAND, Boston, Massachusetts

## Abstract

**Importance:**

Medicare beneficiaries with Alzheimer disease and related dementias (ADRD) are a particularly vulnerable group in whom arthritis is a frequently occurring comorbidity. Medicare’s mandatory bundled payment reform—the Comprehensive Care for Joint Replacement (CJR) model—was intended to improve quality and reduce spending in beneficiaries undergoing joint replacement surgical procedures for arthritis. In the absence of adjustment for clinical risk, hospitals may avoid performing elective joint replacements for beneficiaries with ADRD.

**Objective:**

To evaluate the association of the CJR model with utilization of joint replacements for Medicare beneficiaries with ADRD.

**Design, Setting, and Participants:**

This cohort study used national Medicare data from 2013 to 2017 and multivariable linear probability models and a triple differences estimation approach. Medicare beneficiaries with a diagnosis of arthritis were identified from 67 metropolitan statistical areas (MSAs) mandated to participate in CJR and 104 control MSAs. Data were analyzed from July 2020 to July 2021.

**Exposures:**

Implementation of the CJR model in 2016.

**Main Outcomes and Measures:**

Outcomes were separate binary indicators for whether or not a beneficiary underwent hip or knee replacement. Key independent variables were the MSA group, before-CJR and after-CJR phase, ADRD diagnosis, and their interactions. The linear probability models controlled for beneficiary characteristics, MSA fixed effects, and time trends.

**Results:**

The study included 24 598 729 beneficiary-year observations for 9 624 461 unique beneficiaries, of which 250 168 beneficiaries underwent hip and 474 751 underwent knee replacements. The mean (SD) age of the 2013 cohort was 77.1 (7.9) years, 3 110 922 (66.4%) were women, 3 928 432 (83.8%) were non-Hispanic White, 792 707 (16.9%) were dually eligible for Medicaid, and 885 432 (18.9%) had an ADRD diagnosis. Before CJR implementation, joint replacement rates were lower among beneficiaries with ADRD (hip replacements: 0.38% vs 1.17% for beneficiaries with and without ADRD, respectively; *P* < .001; knee replacements: 0.70% vs 2.25%; *P* < .001). After controlling for relevant covariates, CJR was associated with a 0.07-percentage-point decline in hip replacements for beneficiaries with ADRD (95% CI, −0.13 to −0.001; *P* = .046) and a 0.07-percentage-point decline for beneficiaries without ADRD (95% CI, −0.12 to −0.02; *P* = .01) residing in CJR MSAs compared with beneficiaries in control MSAs. However, this change in hip replacement rates for beneficiaries with ADRD was not statistically significantly different from the change for beneficiaries without ADRD (percentage point difference: 0.01; 95% CI, −0.08 to 0.09; *P* = .88). No statistically significant changes in knee replacement rates were noted for beneficiaries with ADRD compared with those without ADRD with CJR implementation (percentage point difference: −0.03, 95% CI, −0.09 to 0.02; *P* = .27).

**Conclusions and Relevance:**

In this cohort study of Medicare beneficiaries with arthritis, the CJR model was not associated with a decline in joint replacement utilization among beneficiaries with ADRD compared with beneficiaries without ADRD in the first 2 years of the program, thereby alleviating patient selection concerns.

## Key Points

**Question:**

Is the Comprehensive Care for Joint Replacement (CJR) model associated with changes in hip and knee replacement use for Medicare beneficiaries with Alzheimer disease and related dementias (ADRD)?

**Findings:**

In this cohort study of 24 598 729 beneficiary-year observations among 9 624 461 unique beneficiaries, CJR was statistically significantly associated with a decrease in hip replacement use for beneficiaries with and without ADRD; however, the gap in use between these groups did not change with CJR implementation. The CJR model was not associated with changes in knee replacement use.

**Meaning:**

This study found that the CJR model was not associated with a disproportionate reduction in joint replacement use for Medicare beneficiaries with ADRD.

## Teaser

This cohort study evaluates the association of the Comprehensive Care for Joint Replacement model with utilization of joint replacements for Medicare beneficiaries with Alzheimer disease and related dementias.

## Introduction

Alzheimer disease and related dementias (ADRD) are among the leading causes of morbidity and mortality among older adults,^1^ making the 6.2 million older individuals in the US with ADRD a particularly vulnerable and high-priority population.^2^ The physical and cognitive impairments due to ADRD progressively increase and severely limit a patient’s ability to carry out routine activities, placing them at an increased risk of health care use and death and imposing an unprecedented demand on their families and caregivers. In 2021, Medicare alone financed more than 50% of the $355 billion that was spent on ADRD care, with hospitalizations, emergency department use, and postacute care being the main drivers of this spending.^1^

Because increasing age is an important risk factor for ADRD^3^ and arthritis is a frequently occurring comorbidity,^4^ patients with ADRD are likely to need arthritis treatments, including the use of elective joint replacements (total hip [THR] and total knee replacements [TKR]). These surgeries are highly effective in alleviating pain, improving physical function, and enhancing health-related quality of life.^5^ Because joint replacements are among the most frequently performed inpatient surgical procedures for older Medicare beneficiaries,^6^ and there is considerable variability in the outcomes and spending for these procedures, joint replacements are included in several payment reforms launched by the Centers for Medicare & Medicaid Services (CMS). One such reform is the Comprehensive Care for Joint Replacement (CJR) model, which was mandated for most hospitals located in 67 randomly selected metropolitan statistical areas (MSAs) in 2016.^7^ The CJR bundles spending for the joint replacement episode, which includes the inpatient stay and 90-day postacute care period. Hospitals are accountable for maintaining the episode spending below the quality-adjusted spending benchmarks. Depending on their performance, hospitals can earn reconciliation payments or are required to repay CMS. Although the CJR model was found to modestly reduce joint replacement spending without compromising quality,^8^ it was also found to widen the gap in TKR use between White and Black Medicare beneficiaries.^9,10^

The CJR model’s potential to limit access to joint replacements for vulnerable patients may be particularly relevant for beneficiaries with ADRD. Although the CJR model adjusts its spending benchmarks using the Medicare Severity Diagnosis Related Groups and diagnosis of fractures, it does not explicitly account for the clinical and social risk of patients. Hence, to achieve higher performance on CJR metrics, hospitals may selectively avoid elective surgery for beneficiaries with ADRD who have disproportionately higher clinical needs, a higher risk of adverse events, and increased spending. Thus, Medicare beneficiaries with ADRD, especially those with arthritis, are vulnerable and at an increased risk of being discriminated against or encountering barriers to the use of joint replacements compared with beneficiaries without ADRD.

The objective of our study was to examine the association of the CJR model with the utilization of elective THRs and TKRs for Medicare beneficiaries with and without ADRD. We hypothesize that a substantial gap exists in joint replacement use between beneficiaries with and without ADRD and that the CJR was associated with a greater decline in the use of these procedures for beneficiaries with ADRD, thereby widening the gap in joint replacement use between the 2 groups. Our study is the first to our knowledge to investigate whether mandatory bundled payment reforms such as the CJR model are likely to be associated with a decline in joint replacement use for Medicare beneficiaries with ADRD, thereby providing vital evidence regarding the role of such reforms in creating barriers to health care use and access for beneficiaries with ADRD.

## Methods

### Data Sources and Study Cohort

We obtained demographic and enrollment data of all Medicare beneficiaries from the 2013 to 2017 Medicare Master Beneficiary Summary Files (MBSF).^11^ We used these files to identify Medicare beneficiaries who met our inclusion criteria and lived in the 67 MSAs mandated to participate in the CJR program or the 104 control MSAs (eAppendix 1 in the Supplement). The MSAs were randomized to treatment and control groups by Medicare.^7^ Although 75 MSAs were originally assigned to the treatment group, 8 MSAs were excluded from participation at the start of the CJR implementation mostly owing to changes in participation in other payment reforms.^12^ We used the MBSF-Chronic Conditions (MBSF-CC) segment to identify chronic conditions among Medicare beneficiaries. These chronic conditions are determined by Medicare using validated algorithms and claims data.^13^ We limited the cohort to beneficiaries who met the claims criteria for rheumatoid arthritis or osteoarthritis in a given data year because these beneficiaries are most likely to be at risk/eligible for THRs/TKRs.

The Medicare Provider Analysis and Review files contain details about inpatient and skilled nursing facility stays covered by Medicare.^14^ We used CJR-specific and other inclusion criteria to identify stays for elective THRs and TKRs (eAppendix 1 in the Supplement). We excluded hip fracture stays from the cohort because hospitals are unlikely to discriminate between patients in nonelective situations. We excluded stays in hospitals that were not reimbursed by the Inpatient Prospective Payment System and hospitals that participated in the Bundled Payments for Care Improvement program. The main analytic cohort consisted of 24 598 729 beneficiary-year observations from 2013 to 2017 for 9 624 461 unique beneficiaries with arthritis, of which 250 168 underwent THRs and 474 751 underwent TKRs. The study was approved and granted a waiver of participant informed consent by the University of Rochester Research Subject Review Board.

### Key Variables

The outcomes were separate binary indicators for whether a beneficiary underwent a THR or TKR during a given year. The study had 3 key independent variables. First was a binary indicator of whether a beneficiary resided in an MSA that was mandated to participate in the CJR or in a control MSA. The second variable was the before-CJR or after-CJR phase with the before-CJR phase extending from 2013 to 2015 and the after-CJR phase being 2017. We excluded observations from 2016 in the main multivariable analysis because CJR’s implementation in April 2016 does not permit the classification of beneficiaries in 2016 into a before-CJR or after-CJR cohort. Third was a binary indicator of whether or not a beneficiary was ever diagnosed with ADRD.^15,16,17^ This indicator was determined by Medicare using ADRD diagnosis codes in multiple claims data sources. In statistical analysis, we included the main effects of these 3 variables along with their 2-way and 3-way interactions.

The multivariable models controlled for continuous specifications of age and calendar year and categorical specifications of sex, race and ethnicity (Asian, Hispanic, non-Hispanic Black, non-Hispanic White, North American Native, unknown, other), dual eligibility, and 23 chronic conditions. Race and ethnicity data were obtained from the MBSF, which is populated from Social Security Administration records.^18^

### Statistical Analysis

We report means (and SDs) for continuous variables and numbers (and percentages) for categorical variables. We used Kruskal-Wallis and χ^2^ tests to test differences in the distribution of variables across treatment and control MSAs. We estimated multivariable linear probability models with MSA-level fixed effects and Huber-White robust sandwich estimators of variance. These models tested whether CJR was associated with higher or lower THR/TKR use for beneficiaries with ADRD compared with those without ADRD. We used a triple differences estimation approach to determine this association.^9,19^ Before estimating these models, we tested for the parallel trends assumption, which examines trends in surgical use for beneficiaries with and without ADRD before CJR implementation. In case of violation of the assumption, we included interactions of the year with CJR treatment/control MSA and ADRD indicators. We used Lewin Group’s methods to determine MSA-level weights, which accounted for selection probabilities of the treatment and control MSAs.

All analyses were performed using Stata/MP version 16.1 (Unix) (StataCorp LLC). A 2-tailed *P* < .05 was considered statistically significant. Details of the analytic and weighting approaches and the null and alternate hypotheses are provided in eAppendixes 2 and 3 in the Supplement.

### Sensitivity Analysis

First, we included all eligible beneficiaries and did not limit the cohort to beneficiaries with arthritis. Second, we reestimated the models using observations from the 75 treatment MSAs originally mandated to participate in the CJR program (intention-to-treat analysis) and the corresponding 121 control MSAs. Third, we defined elective surgical procedures using Medicare’s criteria for calculating risk-standardized complication and readmission rates for joint replacements.^20^ Fourth, we limited the cohort to beneficiaries diagnosed with ADRD within the past year because these beneficiaries are likely to derive greater value from joint replacements than those with long-standing ADRD. Fifth, we reestimated the models among those with Alzheimer disease only instead of those with ADRD. Sixth, we estimated logistic regression models instead of linear probability models to determine the sensitivity of the main findings to model specifications. Finally, we included observations from 2016 into the after-CJR phase because hospitals may have introduced changes before the start of the CJR in April 2016.

We followed the Strengthening the Reporting of Observational Studies in Epidemiology (STROBE) reporting guideline to report this study.^21^ Data were analyzed from July 2020 to July 2021.

## Results

### Descriptive Statistics

The cohort in 2013 included 4 688 663 Medicare beneficiaries from 67 CJR MSAs and 104 control MSAs (Table 1). Of these beneficiaries, 885 432 (18.9%) had a diagnosis of ADRD, with ADRD prevalence being 20.1% in CJR MSAs and 17.9% in control MSAs (*P* < .001). Overall, the mean (SD) age was 77.1 (7.9) years, 3 110 922 (66.4%) were women, 3 928 432 (83.8%) were non-Hispanic White, and 792 707 (16.9%) were dually eligible for Medicaid. The mean (SD) number of comorbidities (excluding ADRD) was 4.4 (2.5).

**Table 1.** Descriptive Statistics for Medicare Beneficiaries With a Diagnosis of Arthritis Residing in CJR and Non-CJR MSAs in 2013

Characteristic	No. (%)			*P* value^a^
	MSAs		Total	
	CJR	Non-CJR		
No.				
MSAs	67	104	171	NA
Beneficiaries^b^	2 167 927	2 520 736	4 688 663	NA
ADRD	434 636 (20.1)	450 796 (17.9)	885 432 (18.9)	<.001
Age, mean (SD), y	77.39 (8.0)	76.92 (7.9)	77.14 (7.9)	<.001
Sex				
Female	1 447 787 (66.8)	1 663 135 (66.0)	3 110 922 (66.4)	<.001
Male	720 140 (33.2)	857 601 (34.0)	1 577 741 (33.7)	
Race and ethnicity				
Asian	84 977 (3.9)	46 846 (1.9)	131 823 (2.8)	<.001
Hispanic	74 778 (3.5)	37 248 (1.5)	112 026 (2.4)	
Non-Hispanic				
Black	174 683 (8.1)	232 064 (9.2)	406 747 (8.7)	
White	1 775 896 (81.9)	2 152 536 (85.4)	3 928 432 (83.8)	
North American Native	3859 (0.2)	6791 (0.3)	10 650 (0.2)	
Unknown	14 998 (0.7)	12 155 (0.5)	27 153 (0.6)	
Other^c^	38 736 (1.8)	33 096 (1.3)	71 832 (1.5)	
Dual eligible	442 512 (20.4)	350 195 (13.9)	792 707 (16.9)	<.001
Sum of comorbidities, mean (SD)^d^	4.49 (2.6)	4.27 (2.5)	4.37 (2.5)	<.001
Replacements^e^				
Hip	20 935 (1.0)	26 215 (1.0)	47 150 (1.0)	<.001
Knee	42 294 (2.0)	55 671 (2.2)	97 965 (2.1)	<.001

Abbreviations: ADRD, Alzheimer disease and related dementias; CJR, Comprehensive Care for Joint Replacement model; MSA, metropolitan statistical area; NA, not applicable.

^a^ *P* values for Kruskal-Wallis tests (for continuous variables) or χ^2^ tests (for categorical variables) that test for the distribution of characteristics across CJR and non-CJR MSAs.

^b^ Data from the 2013 Master Beneficiary Summary File—Base Segment and Chronic Conditions Segment.

^c^ Other: This category is included in Medicare’s Master Beneficiary Summary File.

^d^ Mean of 23 chronic conditions excluding ADRD obtained from the Master Beneficiary Summary File—Chronic Conditions Segment. Distribution of comorbidities for Medicare beneficiaries included in this table is presented in eTable 1 in the Supplement.

^e^ Data from the 2013 Medicare Provider Analysis and Review file. Descriptive statistics for patients who underwent hip and knee replacement are presented in eTables 2 and 3 in the Supplement.

In 2013, the overall THR rate was 1.0%, with the rate being lower in CJR MSAs compared with control MSAs (1.0% vs 1.0%; *P* < .001) (Table 1). The overall TKR rate was 2.1%, with the rate being lower in CJR MSAs compared with control MSAs (2.0% vs 2.2%; *P* < .001). Additional descriptive statistics comparing cohorts in CJR and non-CJR MSAs are presented in eTables 1-3 in the Supplement, and eTable 4 in the Supplement compares beneficiaries with and without ADRD.

### Surgery Rates for Beneficiaries With and Without ADRD

**Figure 1.**
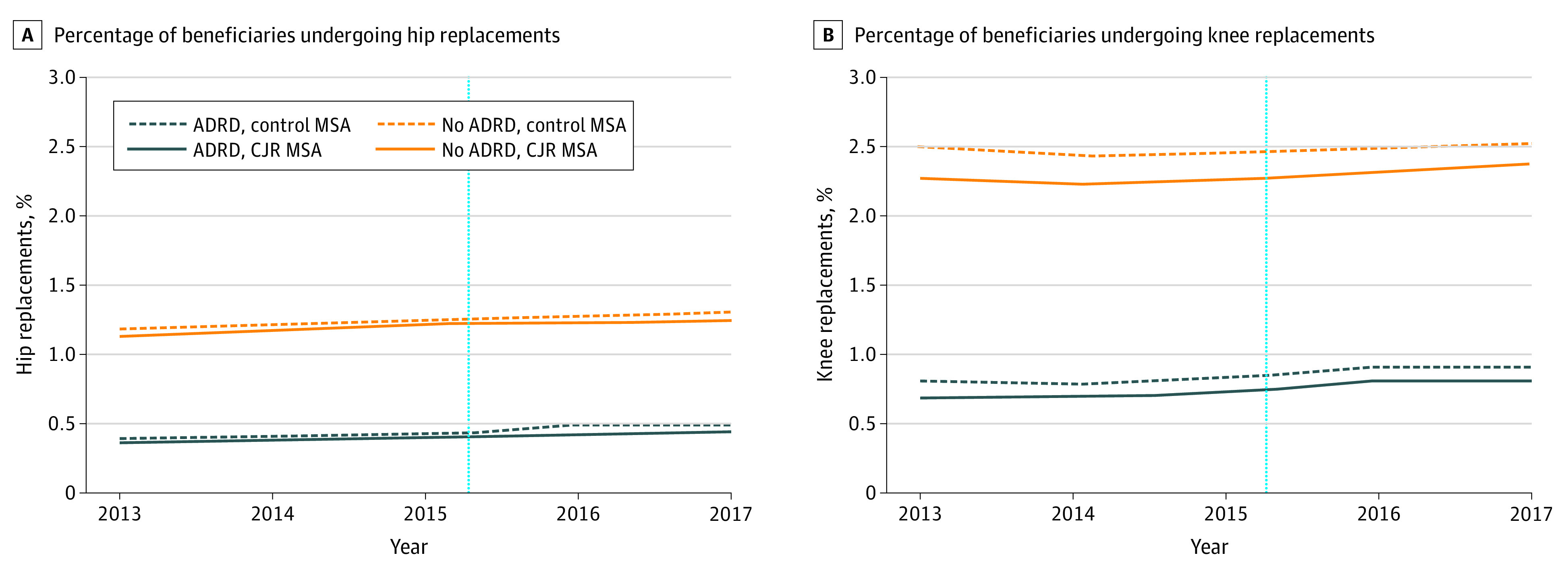
Unadjusted Trends in the Percentage of Medicare Beneficiaries Undergoing Hip and Knee Replacements in CJR and Non-CJR MSAs From 2013 to 2017 ADRD indicates Alzheimer disease and related dementias; CJR, Comprehensive Care for Joint Replacement model; MSA, metropolitan statistical area. Authors’ analysis of the 2013 to 2017 Medicare Master Beneficiary Summary File (base and chronic conditions segment) and Medicare Provider Analysis and Review File. The year markings on the x-axis represent the end of the respective year. The dotted vertical line represents the date of CJR implementation in April 2016.

Before CJR implementation, THR rates for beneficiaries with and without ADRD were 0.38% (95% CI, 0.36% to 0.39%) and 1.17% (95% CI, 1.16% to 1.18%), respectively, in CJR MSAs (Figure 1 and Table 2, column A). This THR rate for beneficiaries with ADRD was 0.79 percentage points lower (95% CI, −0.81 to −0.77; *P* < .001) compared with the rate for beneficiaries without ADRD. Similarly, TKR rates for beneficiaries with and without ADRD were 0.70% (95% CI, 0.67% to 0.72%) and 2.25% (95% CI, 2.23% to 2.26%), respectively, in CJR MSAs. This TKR rate for beneficiaries with ADRD was 1.55 percentage points lower (95% CI, −1.58 to −1.52; *P* < .001) than beneficiaries without ADRD. Similar gaps in surgery use were noted in the period after CJR implementation in CJR MSAs, and among control MSAs.

**Table 2.** Surgical Rates (Expressed in Percentages) for Total Hip and Total Knee Replacement With CJR Implementation (2013-2017) (Observations, n = 19 468 093; MSAs, n = 171)

Surgical procedure	MSA, unadjusted, % (95% CI)				Adjusted, difference % (95% CI)^a^	
	CJR		Non-CJR		Change in CJR MSAs vs non-CJR MSAs	Change with respect to beneficiaries without ADRD
	Column A^b^		Column B^c^		Column C^d^	Column D^e^
	Before	After	Before	After	%-Point difference	%-Point difference
**Hip replacements**						
ADRD status						
ADRD	0.38 (0.36 to 0.39)	0.43 (0.40 to 0.46)	0.41 (0.39 to 0.43)	0.50 (0.47 to 0.53)	−0.07 (−0.13 to −0.001)^f^	0.01 (−0.08 to 0.09)
No ADRD	1.17 (1.16 to 1.18)	1.25 (1.24 to 1.27)	1.21 (1.21 to 1.22)	1.30 (1.29 to 1.31)	−0.07 (−0.12 to −0.02)^g^	1 [Reference]
**Knee replacements**						
ADRD status						
ADRD	0.70 (0.67 to 0.72)	0.80 (0.76 to 0.84)	0.81 (0.78 to 0.83)	0.92 (0.88 to 0.96)	−0.01 (−0.05 to 0.04)	−0.03 (−0.09 to 0.02)
No ADRD	2.25 (2.23 to 2.26)	2.37 (2.35 to 2.39)	2.47 (2.46 to 2.48)	2.52 (2.50 to 2.54)	0.02 (−0.01 to 0.06)	1 [Reference]

Abbreviations: ADRD, Alzheimer disease and related dementias; CJR, Comprehensive Care for Joint Replacement model; MSA, metropolitan statistical area.

^a^ Adjusted rates (expressed in percentages) from patient-level multivariable linear regression models with robust/sandwich estimators of variance. The models assessed CJR’s association with the use of surgical procedures for beneficiaries with and without ADRD in CJR MSAs vs non-CJR MSAs. The models controlled for age, sex, race and ethnicity, dual eligibility, comorbidities, calendar year (and relevant interactions with CJR MSA and ADRD indicator), MSA fixed effects, and MSA weights. The analysis excluded data from 2016 because the CJR was introduced in April 2016, and this implementation precludes the classification of Medicare beneficiaries into a before-CJR and after-CJR cohort.

^b^ Column A presents the unadjusted rates (expressed in percentages) of surgical procedures in CJR MSAs.

^c^ Column B presents the unadjusted rates (expressed in percentages) of surgical procedures in non-CJR MSAs.

^d^ Column C presents the percentage point differences in the rates of surgical procedures for each ADRD status group in CJR MSAs with CJR implementation vs non-CJR MSAs (“double difference”).

^e^ Column D presents the percentage point differences in the rates of surgical procedures for beneficiaries with ADRD compared with those without ADRD in CJR MSAs with CJR implementation vs non-CJR MSAs (“triple difference”).

^f^ *P* = .046.

^g^ *P* = .007.

### Multivariable Analysis

The parallel trends assumption was violated only for the THR cohort (eTable 5 in the Supplement). After controlling for relevant covariates, the CJR model was associated with a 0.07-percentage-point decline in THR use for beneficiaries with ADRD (95% CI, −0.13 to −0.001; *P* = .046) and a 0.07-percentage-point decline for beneficiaries without ADRD (95% CI, −0.12 to −0.02; *P* = .01) residing in CJR MSAs compared with beneficiaries residing in control MSAs (Table 2, column C). However, the decline for beneficiaries with ADRD was not statistically significantly different from the decline for beneficiaries without ADRD (percentage point difference, 0.01; 95% CI, −0.08 to 0.09; *P* = .88) (Table 2, column D), indicating that the gap in THR use between these 2 groups did not change with CJR implementation. Despite this statistically nonsignificant finding, the change in THR use was consistent with effect sizes ranging from a −0.08-percentage-point difference (decline in THR use) to a 0.09-percentage-point difference (increase in THR use) for Medicare beneficiaries with ADRD compared with those without ADRD.

**Figure 2.**
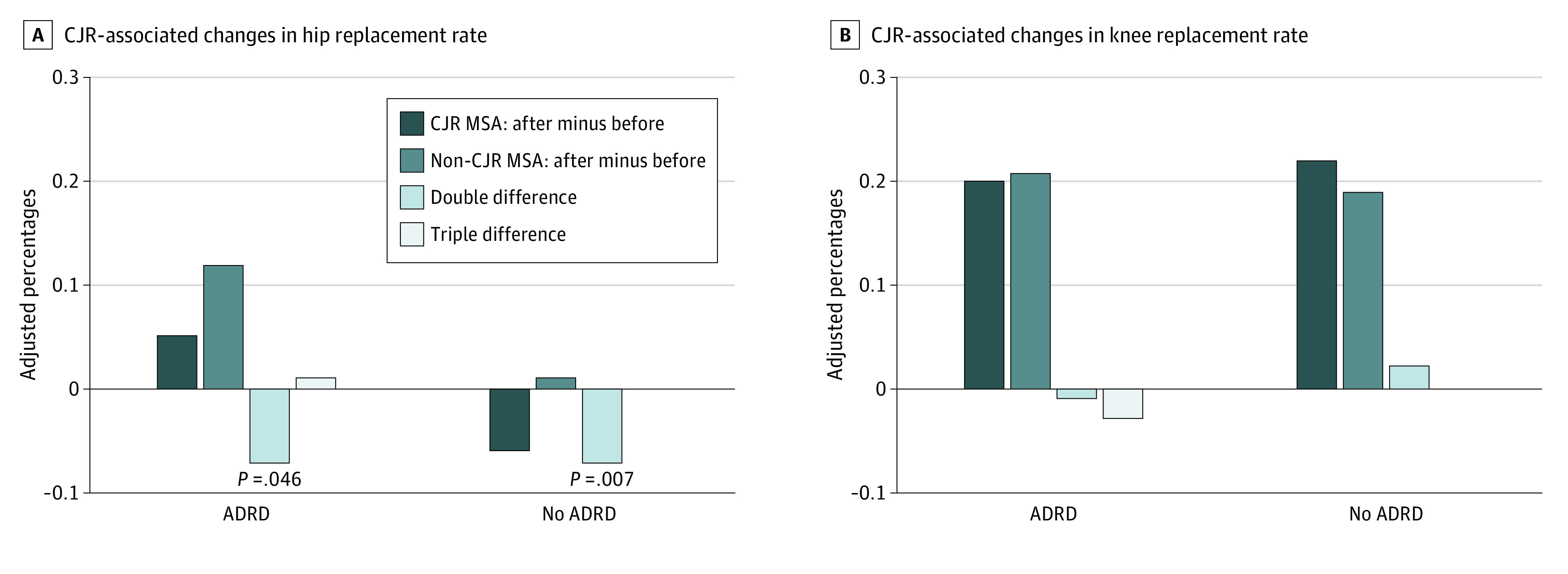
Changes in Adjusted Percentages of Hip and Knee Replacement Use With Comprehensive Care for Joint Replacement Model (CJR) Implementation ADRD indicates Alzheimer disease and related dementias; MSA, metropolitan statistical area. Differences in adjusted percentages derived from patient-level multivariable linear regression models with robust/sandwich estimators of variance (Table 2). The interpretation of the bars are as follows: (a) The CJR MSA bars represent the change in THR/TKR use with CJR implementation in CJR MSAs. (b) The non-CJR MSA bars represent the change in THR/TKR use in non-CJR MSAs. (c) The double difference bars represent the change in THR/TKR use in CJR MSAs compared with non-CJR MSAs (a − b) for beneficiaries with and without ADRD. These are the double differences estimates reported in column C of Table 2. (d) The triple difference bars represent the change in THR/TKR use in CJR MSAs vs non-CJR MSAs for beneficiaries with ADRD compared with those without ADRD (c for ADRD − c for non-ADRD). These estimates are the triple differences estimates reported in column D of Table 2.

The CJR model was not associated with statistically significant changes in TKR use for beneficiaries with (percentage point change, −0.01; 95% CI, −0.05 to 0.04; *P* = .78) or without ADRD (percentage point change, 0.02; 95% CI, −0.01 to 0.06; *P* = .15). The change for beneficiaries with ADRD did not statistically significantly differ from the change for beneficiaries without ADRD (percentage point difference, −0.03; 95% CI, −0.09 to 0.02; *P* = .27). Despite this statistically nonsignificant finding, the change in TKR use was consistent with effect sizes ranging from a −0.09-percentage-point difference (decline in TKR use) to a 0.02-percentage-point difference (increase in TKR use) for Medicare beneficiaries with ADRD compared with those without ADRD. The adjusted changes in rates are graphically presented in Figure 2, and the regression estimates are presented in eTable 6 in the Supplement.

### Sensitivity Analysis

The inferences from the sensitivity analysis were consistent with the main analysis (eTable 7 in the Supplement). An exception was that in the analysis limiting the ADRD cohort to beneficiaries diagnosed with ADRD in the past year, CJR was found to worsen the gap in TKR use between beneficiary groups (percentage point difference, −0.09; 95% CI, −0.18 to 0.00; *P* = .05).

## Discussion

In this national analysis of Medicare beneficiaries from 2013 to 2017, we found that Medicare’s mandatory bundled payment reform, the CJR model, was associated with an overall decline in THR use for both beneficiaries with and without ADRD. However, the decline for beneficiaries with ADRD was not statistically significantly different from that for beneficiaries without ADRD, indicating that the gap in THR use between the 2 groups remained unchanged 2 years after CJR implementation. We did not find statistically significant changes in TKR use with CJR implementation, nor did the CJR widen the preexisting gap in TKR use between beneficiaries with and without ADRD. In a sensitivity analysis limited to beneficiaries diagnosed with ADRD in the past year, the CJR model was associated with a decline in TKR use for these beneficiaries. However, these findings need to be interpreted with caution and should be viewed as hypothesis generating.

Recent studies have shown that CJR may incentivize hospitals to selectively avoid higher-risk beneficiaries because of the lack of clinical and social risk adjustment.^9,10^ This selection process may result in vulnerable beneficiaries such as racial and ethnic minority groups and low-income beneficiaries facing increasing barriers to undergo these much-needed surgeries. In theory, similar barriers may arise for beneficiaries with ADRD. First, because beneficiaries with ADRD have more significant physical and cognitive deficits, these patients are more likely to require costly institutional postacute care, such as skilled nursing facilities,^22,23^ further increasing spending. Because hospitals in the CJR model are known to control spending primarily by discharging patients to home instead of skilled nursing facilities,^8,24^ this strategy in particular may place patients with ADRD at an increased risk of complications and suboptimal outcomes, further discouraging hospitals and surgeons from operating on patients with ADRD. Second, patients with ADRD undergoing joint replacements have higher rates of mortality^25,26^ and complications such as recurrent dislocation^27,28^ or delirium^29,30^ compared with patients without ADRD. These complications and associated readmissions are likely to be adversely associated with hospital quality and spending performance in the CJR model. Given these mechanisms, our finding that the CJR model did not disproportionately worsen joint replacement use for beneficiaries with ADRD is reassuring.

The present study found that joint replacement use was statistically significantly lower for beneficiaries with ADRD than for those without ADRD. This difference may result from the weakened physical and cognitive status of beneficiaries with ADRD, which places them at an increased risk of adverse events, mortality, and long-term care needs.^25,26^ This increased risk is likely to contribute to decisions by patients, family members, and physicians to avoid surgery. The finding of declines in joint replacement use for both groups of beneficiaries following CJR implementation may result from a careful selection of patients such that quality and spending scores for hospitals are optimized. This finding differs from previous research that examined joint replacement volume as a secondary outcome and did not find statistically significant changes with CJR implementation,^8,24^ and from previous conceptual frameworks that bundled payments may increase hospital joint replacement volume because of increased efficiency resulting from CJR-motivated quality improvement initiatives.^31,32^ The difference in findings is likely explained by the cohort definition, our study design focusing on beneficiary-level instead of aggregate MSA-level joint replacement use, and our granular adjustment for comorbidities. Our finding of a decline in TKR use for beneficiaries recently diagnosed with ADRD is concerning and needs to be further investigated because these surgical procedures may have the greatest value for individuals who are in the early stages of the ADRD disease process.

The study findings have important policy, practice, and research implications. First, in recent years there has been a growing call for CMS to consider more comprehensive risk adjustment of reforms to account for the increased clinical and social risk of vulnerable beneficiaries, such as those with ADRD.^33^ A recent update to the CJR model will now result in adjusting the benchmarks for age, a Hierarchical Condition Category measure of clinical risk, and dual eligibility for Medicaid.^7^ This may alleviate the overall decline in THR use and the decline in TKR use for beneficiaries newly diagnosed with ADRD that we note. Second, although the CJR model and other payment reforms are being evaluated for their potential unintended effects on surgery use among racial and ethnic minority groups and low-income beneficiaries,^9,10^ little attention has been paid to other vulnerable beneficiaries, such as those with ADRD. A greater emphasis on this patient population will ensure that reforms do not create barriers to health care use for those in need of care. Third, the study’s population-level findings are driven by granular decisions made by surgeons and other clinicians. A continual examination of the clinical decision-making process, including goals of care and patient preferences, will be needed to determine the appropriateness of surgery under different scenarios. Finally, further work is needed to monitor and correct the unintended yet likely consequences of not only the CJR but also of other payment reforms.

### Limitations

The present study has several limitations. First, the analysis does not account for unmeasured confounders that may influence the decision to undergo surgery, such as patient preference for surgery or availability of a caregiver, because this information is not available in national Medicare data. Second, we identified the presence of ADRD and arthritis using data from the MBSF-Chronic Conditions segment. Medicare uses rigorously validated algorithms to identify these conditions. However, because of the nature of the data, we were unable to identify the severity or progression of ADRD, and the severity, type, or location of arthritis. These concerns are partly mitigated by reestimating our models among beneficiaries who were newly diagnosed with ADRD and by using data from all Medicare beneficiaries in the sensitivity analysis. Moreover, the prevalence of these conditions is unlikely to change differentially over the study period. Finally, in 2018, the design of the CJR was changed from a fully mandated program to a partially mandated program. Because this program update changes the randomized selection of the MSAs, we limited the analysis to 2017, and we are unable to detect changes that may have occurred more than 2 years after implementation.

## Conclusions

In this cohort study of Medicare beneficiaries with arthritis, we found that the CJR was not associated with a worsening of the gap in joint replacement use between Medicare beneficiaries with and without ADRD. With the onset of the value-based payment approach to health care, CMS has increasingly relied on reforms such as the CJR to improve the quality of care while controlling spending. Despite being well intended, these reforms may unintentionally limit care for vulnerable patient populations. Although the study did not find these unintended effects among beneficiaries with ADRD in the first 2 years of the CJR, continual monitoring of these reforms will ensure that beneficiaries with ADRD and other vulnerable patients receive equitable and effective care.

## Notes

Supplement. **eAppendix 1.** Cohort determination criteria **eAppendix 2.** Key variables, model estimation, and sensitivity analysis **eAppendix 3.** Weighting strategy to account for MSA selection probability **eTable 1.** Chronic Conditions for Medicare beneficiaries with a diagnosis of rheumatoid arthritis/osteoarthritis residing in CJR and non-CJR MSAs in 2013 **eTable 2.** Descriptive statistics for Medicare beneficiaries with a diagnosis of rheumatoid arthritis/osteoarthritis residing in CJR and non-CJR MSAs who underwent hip replacement in 2013 **eTable 3.** Descriptive statistics for Medicare beneficiaries with a diagnosis of rheumatoid arthritis/osteoarthritis residing in CJR and non-CJR MSAs who underwent knee replacement in 2013 **eTable 4.** Descriptive statistics for Medicare beneficiaries with or without a diagnosis of Alzheimer Disease and Related Dementias (ADRD) in 2013 **eTable 5.** Adjusted estimates for parallel trends test from multivariable models examining the use of hip and knee replacement surgeries before the Comprehensive Care for Joint Replacement model was implemented (2013-2015) **eTable 6.** Full model estimates from multivariable models examining the use hip or knee replacement surgeries before and after the Comprehensive Care for Joint Replacement model was implemented (2013-2017) **eTable 7.** Sensitivity Analyses - The Comprehensive Care for Joint Replacement model’s association with use of hip and knee replacements for beneficiaries with ADRD compared to beneficiaries without ADRD (2013-2017)
eReferences
