# Supplementary material for: Association of Medicare Mandatory Bundled Payment Reform With Joint Replacement Surgery Use for Beneficiaries With Alzheimer Disease and Related Dementias
Source: JAMA Health Forum. 2022 Feb 11;3(2):e215111. doi: 10.1001/jamahealthforum.2021.5111 (PMC8903111; doi:10.1001/jamahealthforum.2021.5111)
Supplement: Supplement. — eAppendix 1. Cohort determination criteria eAppendix 2. Key variables, model estimation, and sensitivity analysis eAppendix 3. Weighting strategy to account for MSA selection probability eTable 1. Chronic Conditions for Medicare beneficiaries with a diagnosis of rheumatoid arthritis/osteoarthritis residing in CJR and non-CJR MSAs in 2013 eTable 2. Descriptive statistics for Medicare beneficiaries with a diagnosis of rheumatoid arthritis/osteoarthritis residing in CJR and non-CJR MSAs who underwent hip replacement in 2013 eTable 3. Descriptive statistics for Medicare beneficiaries with a diagnosis of rheumatoid arthritis/osteoarthritis residing in CJR and non-CJR MSAs who underwent knee replacement in 2013 eTable 4. Descriptive statistics for Medicare beneficiaries with or without a diagnosis of Alzheimer Disease and Related Dementias (ADRD) in 2013 eTable 5. Adjusted estimates for parallel trends test from multivariable models examining the use of hip and knee replacement surgeries before the Comprehensive Care for Joint Replacement model was implemented (2013-2015) eTable 6. Full model estimates from multivariable models examining the use hip or knee replacement surgeries before and after the Comprehensive Care for Joint Replacement model was implemented (2013-2017) eTable 7. Sensitivity Analyses - The Comprehensive Care for Joint Replacement model’s association with use of hip and knee replacements for beneficiaries with ADRD compared to beneficiaries without ADRD (2013-2017) eReferences [file jamahealthforum-e215111-s001.pdf]

Supplemental Online Content

Thirukumaran CP, Ricciardi BF, Cai X, Holloway RG, Li Y, Glance LG. Association of Medicare mandatory bundled payment reform with joint replacement surgery use for beneficiaries with Alzheimer disease and related dementias. *JAMA Health Forum*. 2022;3(2):e215111. doi:10.1001/jamahealthforum.2021.5111

eAppendix 1. Cohort determination criteria

eAppendix 2. Key variables, model estimation, and sensitivity analysis

eAppendix 3. Weighting strategy to account for MSA selection probability

eTable 1. Chronic Conditions for Medicare beneficiaries with a diagnosis of rheumatoid arthritis/osteoarthritis residing in CJR and non-CJR MSAs in 2013

eTable 2. Descriptive statistics for Medicare beneficiaries with a diagnosis of rheumatoid arthritis/osteoarthritis residing in CJR and non-CJR MSAs who underwent hip replacement in 2013

eTable 3. Descriptive statistics for Medicare beneficiaries with a diagnosis of rheumatoid arthritis/osteoarthritis residing in CJR and non-CJR MSAs who underwent knee replacement in 2013

eTable 4. Descriptive statistics for Medicare beneficiaries with or without a diagnosis of Alzheimer Disease and Related Dementias (ADRD) in 2013

eTable 5. Adjusted estimates for parallel trends test from multivariable models examining the use of hip and knee replacement surgeries before the Comprehensive Care for Joint Replacement model was implemented (2013-2015)

eTable 6. Full model estimates from multivariable models examining the use hip or knee replacement surgeries before and after the Comprehensive Care for Joint Replacement model was implemented (2013-2017)

eTable 7. Sensitivity Analyses - The Comprehensive Care for Joint Replacement model's association with use of hip and knee replacements for beneficiaries with ADRD compared to beneficiaries without ADRD (2013-2017)

eReferences

This supplemental material has been provided by the authors to give readers additional information about their work.

© 2022 Thirukumaran CP et al. *JAMA Health Forum.*

eAppendix 1. Cohort determination criteria

<u>Inclusion criteria using the Medicare Master Beneficiary Summary (MBSF) File (2013-2017)</u>

- Beneficiaries residing in CJR and non-CJR MSAs
- Fee-for-service beneficiaries identified as those with fee-for-service Medicare coverage for 12 months in the year
- Beneficiaries between 65-99 years of age
- Old age and not end-stage renal disease as a reason for Medicare entitlement
- Beneficiaries who were alive at the end of the calendar year
- Beneficiaries with no missing data for race/ethnicity, dual-eligibility, or sex

Beneficiaries were attributed to the MSA determined using their zip code at the end of the year.

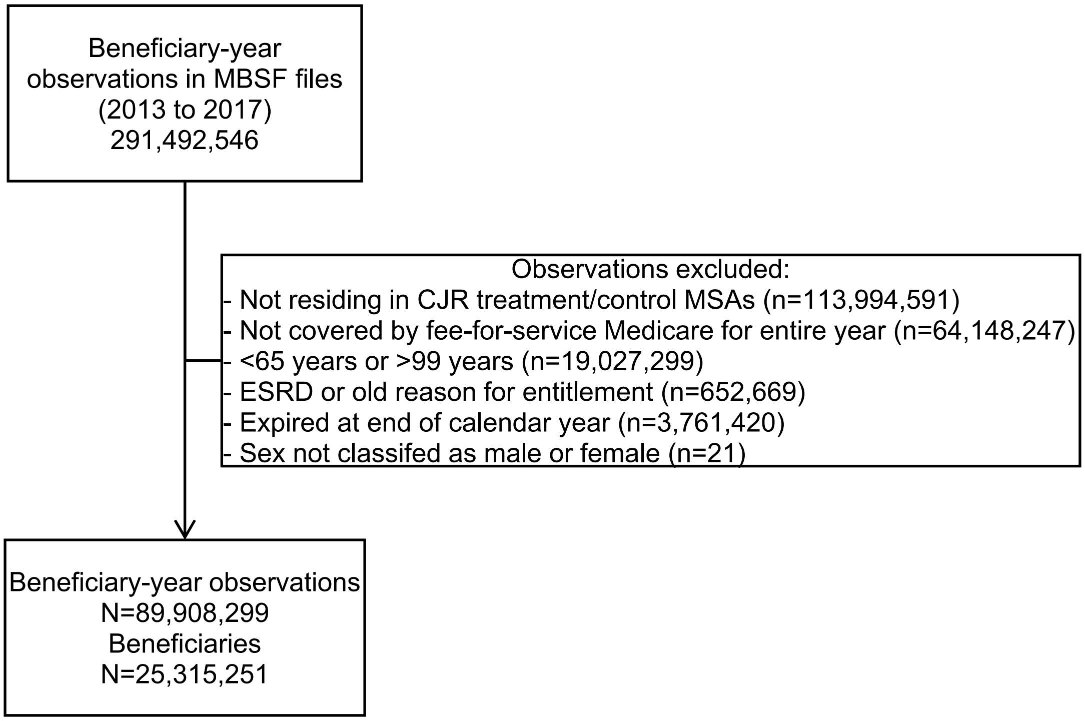

<u>Inclusion criteria using the Medicare Provider Analysis and Review file (MedPAR) (2013-2017)</u>

- Medicare Severity Diagnosis Related Group (MS-DRG) codes 469 and 470
- Meet MBSF inclusion criteria listed above
- Short, inpatient stays
- Hospitals reimbursed by the Inpatient Prospective Payment System
- Medicare as the primary payor
- Alive during the 90-day episode
- Non-fracture related, elective stays
- Enrolled in Parts A and B of Medicare at the time of admission
- Hospitals not participating in Model 1 or the risk-bearing phase of Models 2 and 3 of the Bundled Payments for Care Improvement initiative
- Inpatient stays for total hip replacement and total knee replacement
- Non-duplicate inpatient stays
- Qualifying stays that do not occur during the 90-day episode of a previous qualifying stay

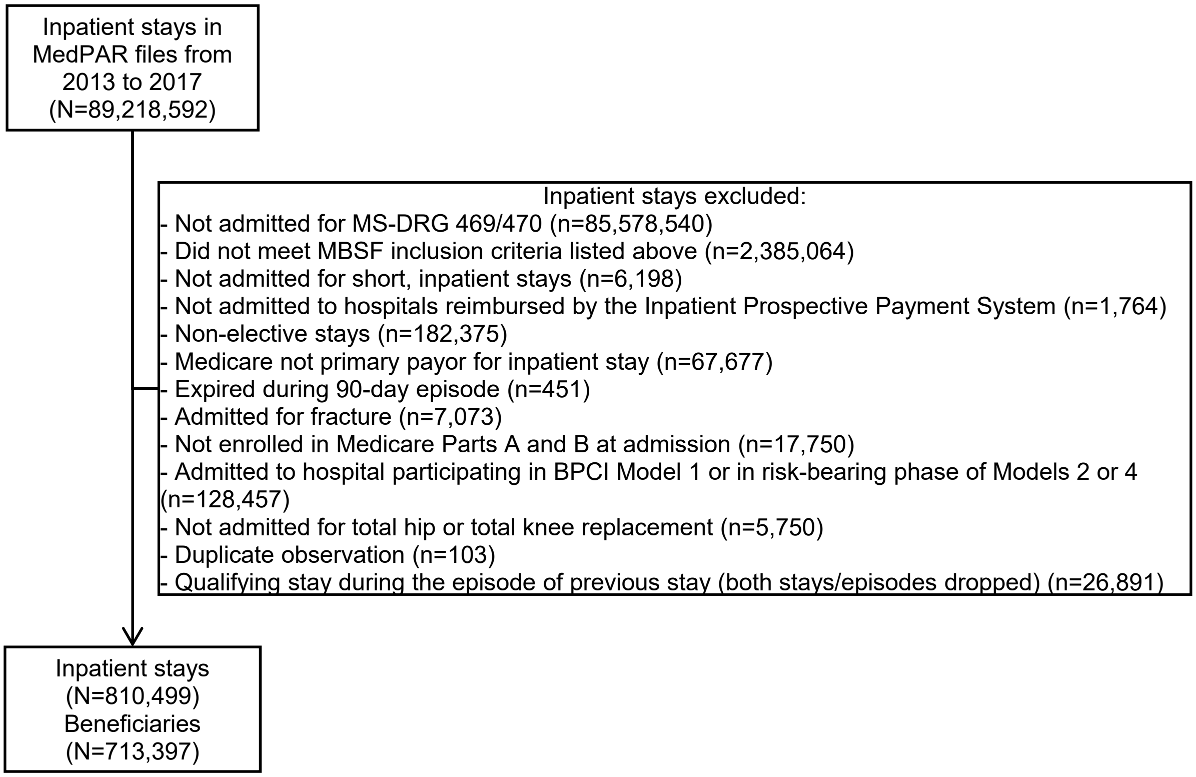

© 2022 Thirukumaran CP et al. *JAMA Health Forum.*

Inclusion criteria from merging the MBSF and MedPAR files (2013-2017)

Note: The files were merged at the beneficiary-level. Two binary indicators (one for hip and one for knee replacements) were created to represent whether a beneficiary had one or more qualifying stay during the year.

- Beneficiaries with joint replacements that meet CJR criteria or beneficiaries who did not undergo any joint replacements
- Beneficiaries with diagnosis of rheumatoid arthritis/osteoarthritis.
- No hip fracture claims

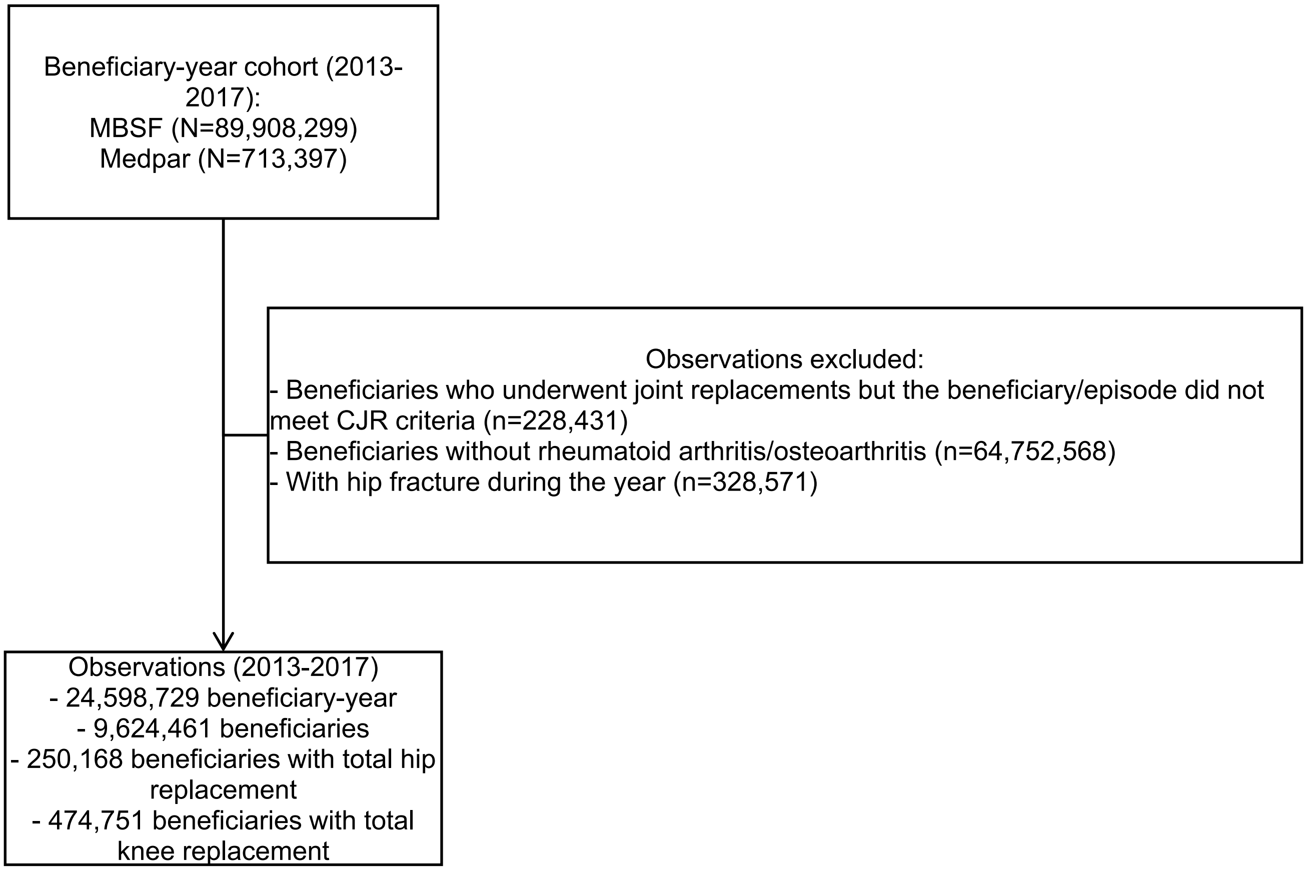

© 2022 Thirukumaran CP et al. *JAMA Health Forum.*

eAppendix 2. Key variables, model estimation, and sensitivity analysis

Hypotheses for parallel trends assumption

Null hypothesis: In the pre-CJR period, the use of THRs/TKRs for beneficiaries with ADRD (compared to those without ADRD) is not statistically significantly different between treatment and control MSAs.

Alternate hypothesis: In the pre-CJR period, the use of THRs/TKRs for beneficiaries with ADRD (compared to those without ADRD) is statistically significantly different between treatment and control MSAs.

Test for parallel trends assumption for triple differences models

To assess whether the trends in the use of joint replacements for Medicare beneficiaries with or without ADRD were parallel in the period before the CJR was implemented (parallel trends assumption for the triple differences models), we estimated the following models (separate for hip and knee replacements). The data for these models was limited to 2013-2015 (pre-CJR period).

$$f\left[E\left(Y_{pmt}\right)\right] = \beta_0 + \beta_1 \times ADRD_{pmt} + \beta_2 \times CJR_m + \beta_3 \times ADRD_{pmt} \times CJR_m + \beta_4 \times Year_t + \beta_5 \times Year_t \times ADRD_{pmt} + \beta_6 \times Year_t \times CJR_m + \beta_7 \times Year_t \times ADRD_{pmt} \times CJR_m + \beta_9 \times X_{pmt} + \beta_{10} MSA_m$$

Y_{pmt}: Binary indicator of whether patient p residing in MSA m in year t underwent hip (or knee) replacement

$f(.)$: Linear identity function

CJR_m: Binary indicator of whether MSA m was a CJR or non-CJR MSA

$Year_t$: Categorical indicator of the year

X_{pmt}: Vector of patient covariates include age, sex, and 25 chronic conditions identified from the MBSF-CC file

© 2022 Thirukumaran CP et al. *JAMA Health Forum.*

MSA_m: Fixed effects of MSA represented using a categorical indicator

$ADRD_{pmt}$: A categorical indicator of ADRD or not.

We used the Wald test to separately test for the statistical significance of β_5, β_6, and β_7. A p-value <0.05 on the test for any of the three estimates in a model represented a violation of the parallel trends assumption.

Results of the parallel trends tests for triple differences models

The parallel trends tests were statistically significant only for hip replacements in the main analysis.

Hypotheses for triple differences model:

Null hypothesis: With CJR implementation, the change in the use of THRs/TKRs for beneficiaries with ADRD (compared to those without ADRD) is not statistically significantly different between treatment and control MSAs.

Alternate hypothesis: With CJR implementation, the change in the use of THRs/TKRs for beneficiaries with ADRD (compared to those without ADRD) is statistically significantly different between treatment and control MSAs.

Model estimation for triple differences models

We estimated the following triple differences models (separate for hip and knee replacements) to assess the differential effect of the CJR. The triple differences approach includes estimating three differences: First, the difference between the pre- and post-CJR rates for each ADRD/non-ADRD group in CJR and non-CJR MSAs (difference 1). Second, the difference in difference 1 between CJR and non-CJR MSAs for each ADRD/non-ADRD group (difference 2). Third, the difference in difference 2 between beneficiaries with ADRD compared to those without ADRD (difference 3).

© 2022 Thirukumaran CP et al. *JAMA Health Forum.*

Because of the violation of the parallel trends assumption, we included interactions of $Year_t$ with $ADRD_{pmt}$ and CJR_m (for the hip replacement models) to account for the differential trends in the pre-CJR period.[1, 2]

$$f\left[E\left(Y_{pmt}\right)\right] = \beta_0 + \beta_1 \times ADRD_{pmt} + \beta_2 \times CJR_m + \beta_3 \times ADRD_{pmt} \times CJR_m + \beta_4 \times Year_t + \beta_5 \times Post_t + \beta_6 \times Post_t \times ADRD_{pmt} + \beta_7 \times Post_t \times CJR_m + \beta_8 \times Post_t \times ADRD_{pmt} \times CJR_m + \beta_9 \times X_{pmt} + \beta_{10} MSA_m + \beta_{11} \times Year_t \times ADRD_{pmt} + \beta_{12} \times Year_t \times CJR_m + \beta_{13} \times Year_t \times ADRD_{pmt} \times CJR_m$$

$Post_t$ is a binary indicator for the CJR implementation phase (Years 2013-2015=0, year 2017=1). The interpretation of other terms is similar to those described previously. β_8 is the triple differences estimate for CJR's association hip/knee replacement use for beneficiaries with ADRD as compared to beneficiaries without ADRD.

We used Stata/MP 16.1 for Unix to estimate the models. We used Stata's margins and lincom commands to test the study hypotheses.

© 2022 Thirukumaran CP et al. *JAMA Health Forum.*

eAppendix 3. Weighting strategy to account for MSA selection probability

We used the method by the Lewin Group to account for an MSA's probability of selection into the treatment (CJR MSAs) or control (non-CJR MSAs) group.[3] In this approach, the CJR MSAs were assigned a weight of 1 and the non-CJR MSAs were assigned weights to represent the CJR MSAs. These weights for the non-CJR MSAs were obtained by dividing the number of CJR MSAs in each of the 8 strata (constructed by the CMS using quartiles of pre-period episode spending and whether the MSA had above or below median population) by the number of non-CJR MSAs in that stratum.

Exhibit E-9: CMS' stratified random sample of CJR MSAs and analytic weights

MSA population	MSA sampling stratum	MSA average episode payment	# MSAs eligible for sampling	CJR sample: # CJR MSAs	CJR sample: Proportion of MSAs selected for CJR	CJR sample: CJR weight	Control group sample: # Control group MSAs	Control group sample: Proportion of MSAs in the control group	Control group sample: Control group weight
Less than median population	1	Lowest quartile	25	8	32.0%	1.0	17	68.0%	8/17
	2	2nd lowest quartile	18	6	33.3%	1.0	12	66.7%	6/12
	3	3rd lowest quartile	19	8	42.1%	1.0	11	57.9%	8/11
	4	Highest quartile	22	11	50.0%	1.0	11	50.0%	11/11
More than median population	5	Lowest quartile	15	5	33.3%	1.0	10	66.7%	5/10
	6	2nd lowest quartile	28	10	35.7%	1.0	18	64.3%	10/18
	7	3rd lowest quartile	22	9	40.9%	1.0	13	59.1%	9/13
	8	Highest quartile	22	10	45.5%	1.0	12	54.5%	10/12
		Total	171	67			104		

Source: Lewin analysis of the Medicare Program Comprehensive Care for Joint Replacement Payment Model for Acute Care Hospitals Furnishing Lower Extremity Joint Replacement Services; A Final Rule by the Centers for Medicare & Medicaid Services, 80 FR 73273 (November 24, 2015) (codified at 42 CFR 510).

Note: MSA = metropolitan statistical area.

Source: Lewin Group, CMS Comprehensive Care for Joint Replacements Model: Performance Year 2 Evaluation Report – Appendices, June 2019, Page 29.

© 2022 Thirukumaran CP et al. *JAMA Health Forum.*

eTable 1. Chronic Conditions for Medicare beneficiaries with a diagnosis of rheumatoid arthritis / osteoarthritis residing in CJR and non-CJR MSAs in 2013

	CJR MSAs	Non-CJR MSAs	Total	p-value[c]
**Beneficiaries[a]**				
N	2,167,927	2,520,736	4,688,663	
Chronic Conditions[b]: (%)				
Acute myocardial infarction	18,409 (0.85)	21,714 (0.86)	40,123 (0.86)	0.15
Anemia	816,200 (37.65)	808,494 (32.07)	1,624,694 (34.65)	<0.001
Asthma	167,301 (7.72)	188,782 (7.49)	356,083 (7.59)	<0.001
Atrial fibrillation	255,003 (11.76)	298,636 (11.85)	553,639 (11.81)	0.005
Benign prostatic hyperplasia	208,414 (9.61)	213,485 (8.47)	421,899 (9.00)	<0.001
Breast cancer	101,522 (4.68)	115,242 (4.57)	216,764 (4.62)	<0.001
Cataract	543,316 (25.06)	611,046 (24.24)	1,154,362 (24.62)	<0.001
Chronic Kidney Disease	454,869 (20.98)	538,214 (21.35)	993,083 (21.18)	<0.001
Chronic Obstructive Pulmonary Disease	312,479 (14.41)	360,419 (14.30)	672,898 (14.35)	<0.001
Colorectal cancer	33,931 (1.57)	38,050 (1.51)	71,981 (1.54)	<0.001
Congestive Heart Failure	451,112 (20.81)	487,287 (19.33)	938,399 (20.01)	<0.001
Depression	442,826 (20.43)	505,636 (20.06)	948,462 (20.23)	<0.001
Diabetes	759,416 (35.03)	799,106 (31.70)	1,558,522 (33.24)	<0.001
Endometrial cancer	9,584 (0.44)	10,487 (0.42)	20,071 (0.43)	<0.001
Glaucoma	323,323 (14.91)	339,793 (13.48)	663,116 (14.14)	<0.001
Hyperlipidemia	1,346,437 (62.11)	1,524,309 (60.47)	2,870,746 (61.23)	<0.001
Hypertension	1,619,670 (74.71)	1,876,351 (74.44)	3,496,021 (74.56)	<0.001
Hypothyroidism	457,063 (21.08)	526,900 (20.90)	983,963 (20.99)	<0.001
Ischemic Heart Disease	900,111 (41.52)	946,776 (37.56)	1,846,887 (39.39)	<0.001
Lung cancer	22,438 (1.03)	25,765 (1.02)	48,203 (1.03)	0.17
Osteoporosis	288,136 (13.29)	289,654 (11.49)	577,790 (12.32)	<0.001
Prostate cancer	83,373 (3.85)	98,570 (3.91)	181,943 (3.88)	<0.001
Stroke/ Transient ischemic attack	117,821 (5.43)	135,967 (5.39)	253,788 (5.41)	0.05

Abbreviations: CJR: Comprehensive Care for Joint Replacement Model; MSA: Metropolitan Statistical Area; ADRD: Alzheimer Disease and Related Dementias; %: Column percentage.

Note: [a] Data from the 2013 Master Beneficiary Summary File – Base and Chronic Conditions Segment; [b] Chronic Conditions present at the end of the year as determined by the claims criteria; [c] p-values for Kruskal-Wallis tests (for continuous variables) or chi-square tests (for categorical variables) that test for the distribution of characteristics across CJR and non-CJR MSAs.

© 2022 Thirukumaran CP et al. *JAMA Health Forum.*

eTable 2. Descriptive statistics for Medicare beneficiaries with a diagnosis of rheumatoid arthritis / osteoarthritis residing in CJR and non-CJR MSAs who underwent hip replacement in 2013

	CJR MSAs	Non-CJR MSAs	Total	p-value[c]
**Patients[a]**				
N	20,935	26,215	47,150	
ADRD: %	1,589 (7.59)	1,785 (6.81)	3,374 (7.16)	0.001
Age in years: Mean (SD)	74.74 (6.66)	74.52 (6.55)	74.62 (6.60)	0.001
Female: %	13,003 (62.11)	16,273 (62.08)	29,276 (62.09)	0.94
Race: %				<0.001
Asian	126 (0.60)	83 (0.32)	209 (0.44)	
Hispanic	115 (0.55)	53 (0.20)	168 (0.36)	
Non-Hispanic Black	955 (4.56)	1,161 (4.43)	2,116 (4.49)	
Non-Hispanic White	19,355 (92.45)	24,468 (93.34)	43,823 (92.94)	
North American Native	31 (0.15)	56 (0.21)	87 (0.18)	
Unknown	178 (0.85)	197 (0.75)	375 (0.80)	
Other[d]	175 (0.84)	197 (0.75)	372 (0.79)	
Dual-eligible: %	983 (4.70)	1,053 (4.02)	2,036 (4.32)	<0.001
Sum of chronic conditions[b]: Mean (SD)	4.78 (2.26)	4.62 (2.22)	4.69 (2.24)	<0.001
Chronic conditions[b]: %				
Acute myocardial infarction	140 (0.67)	178 (0.68)	318 (0.67)	0.89
Anemia	14,593 (69.71)	16,623 (63.41)	31,216 (66.21)	<0.001
Asthma	2,133 (10.19)	2,706 (10.32)	4,839 (10.26)	0.63
Atrial fibrillation	2,484 (11.87)	3,002 (11.45)	5,486 (11.64)	0.16
Benign prostatic hyperplasia	2,950 (14.09)	3,338 (12.73)	6,288 (13.34)	<0.001
Breast cancer	1,302 (6.22)	1,701 (6.49)	3,003 (6.37)	0.23
Cataract	5,349 (25.55)	6,529 (24.91)	11,878 (25.19)	0.11
Chronic Kidney Disease	3,695 (17.65)	4,640 (17.70)	8,335 (17.68)	0.89
Chronic Obstructive Pulmonary Disease	2,685 (12.83)	3,441 (13.13)	6,126 (12.99)	0.33
Colorectal cancer	404 (1.93)	508 (1.94)	912 (1.93)	0.95
Congestive Heart Failure	2,731 (13.05)	3,162 (12.06)	5,893 (12.50)	0.001
Depression	4,463 (21.32)	5,747 (21.92)	10,210 (21.65)	0.11
Diabetes	5,191 (24.80)	6,037 (23.03)	11,228 (23.81)	<0.001
Endometrial cancer	181 (0.86)	225 (0.86)	406 (0.86)	0.94
Glaucoma	2,658 (12.70)	3,125 (11.92)	5,783 (12.27)	0.01
Hyperlipidemia	14,711 (70.27)	18,254 (69.63)	32,965 (69.92)	0.13
Hypertension	16,740 (79.96)	21,076 (80.40)	37,816 (80.20)	0.24
Hypothyroidism	5,019 (23.97)	6,161 (23.50)	11,180 (23.71)	0.23
Ischemic Heart Disease	7,652 (36.55)	8,755 (33.40)	16,407 (34.80)	<0.001
Lung cancer	218 (1.04)	246 (0.94)	464 (0.98)	0.26
Osteoporosis	2,876 (13.74)	3,383 (12.90)	6,259 (13.27)	0.01
Prostate cancer	1,173 (5.60)	1,481 (5.65)	2,654 (5.63)	0.83

© 2022 Thirukumaran CP et al. *JAMA Health Forum.*

	CJR MSAs	Non-CJR MSAs	Total	p-value[c]
Stroke/ Transient ischemic attack	700 (3.34)	773 (2.95)	1,473 (3.12)	0.01

Abbreviations: CJR: Comprehensive Care for Joint Replacement Model; MSA: Metropolitan Statistical Area; N: Number; ADRD: Alzheimer Disease and Related Dementias; %: Column percentage; SD: Standard deviation.

Notes: [a] Data from the 2013 Master Beneficiary Summary File – Base Segment; [b] Data from the 2013 Master Beneficiary Summary File – Chronic Conditions Segment. Sum of chronic conditions excluding ADRD; [c] p-values for Kruskal-Wallis tests (for continuous variables) or chi-square tests (for categorical variables) that test for the distribution of characteristics across CJR and non-CJR MSAs; [d] Other: This category is included in Medicare's Master Beneficiary Summary File.

© 2022 Thirukumaran CP et al. *JAMA Health Forum.*

eTable 3. Descriptive statistics for Medicare beneficiaries with a diagnosis of rheumatoid arthritis / osteoarthritis residing in CJR and non-CJR MSAs who underwent knee replacement in 2013

	CJR MSAs	Non-CJR MSAs	Total	p-value[c]
**Patients[a]**				
N	42,294	55,671	97,965	
ADRD: %	3,020 (7.14)	3,641 (6.54)	6,661 (6.80)	<0.001
Age in years: Mean (SD)	74.09 (6.12)	73.88 (6.07)	73.97 (6.09)	<0.001
Female: %	27,804 (65.74)	35,989 (64.65)	63,793 (65.12)	<0.001
Race: %				<0.001
Asian	762 (1.80)	556 (1.00)	1,318 (1.35)	
Hispanic	708 (1.67)	505 (0.91)	1,213 (1.24)	
Non-Hispanic Black	2,310 (5.46)	2,846 (5.11)	5,156 (5.26)	
Non-Hispanic White	37,556 (88.80)	50,626 (90.94)	88,182 (90.01)	
North American Native	79 (0.19)	133 (0.24)	212 (0.22)	
Unknown	318 (0.75)	329 (0.59)	647 (0.66)	
Other[d]	561 (1.33)	676 (1.21)	1,237 (1.26)	
Dual-eligible: %	3,419 (8.08)	3,298 (5.92)	6,717 (6.86)	<0.001
Sum of chronic conditions[b]: Mean (SD)	4.90 (2.18)	4.74 (2.16)	4.81 (2.17)	<0.001
Chronic conditions[b]: %				
Acute myocardial infarction	197 (0.47)	266 (0.48)	463 (0.47)	0.79
Anemia	27,907 (65.98)	32,830 (58.97)	60,737 (62.00)	<0.001
Asthma	4,989 (11.80)	6,686 (12.01)	11,675 (11.92)	0.31
Atrial fibrillation	4,852 (11.47)	6,284 (11.29)	11,136 (11.37)	0.37
Benign prostatic hyperplasia	5,439 (12.86)	6,807 (12.23)	12,246 (12.50)	0.003
Breast cancer	2,686 (6.35)	3,470 (6.23)	6,156 (6.28)	0.45
Cataract	11,044 (26.11)	14,182 (25.47)	25,226 (25.75)	0.02
Chronic Kidney Disease	7,805 (18.45)	10,143 (18.22)	17,948 (18.32)	0.35
Chronic Obstructive Pulmonary Disease	4,939 (11.68)	6,383 (11.47)	11,322 (11.56)	0.30
Colorectal cancer	646 (1.53)	899 (1.61)	1,545 (1.58)	0.28
Congestive Heart Failure	5,434 (12.85)	6,582 (11.82)	12,016 (12.27)	<0.001
Depression	10,255 (24.25)	13,128 (23.58)	23,383 (23.87)	0.02
Diabetes	13,051 (30.86)	16,661 (29.93)	29,712 (30.33)	0.002
Endometrial cancer	340 (0.80)	447 (0.80)	787 (0.80)	0.99
Glaucoma	5,390 (12.74)	6,904 (12.40)	12,294 (12.55)	0.11
Hyperlipidemia	30,963 (73.21)	40,490 (72.73)	71,453 (72.94)	0.10
Hypertension	35,856 (84.78)	47,239 (84.85)	83,095 (84.82)	0.74
Hypothyroidism	10,807 (25.55)	13,994 (25.14)	24,801 (25.32)	0.14
Ischemic Heart Disease	15,596 (36.88)	18,927 (34.00)	34,523 (35.24)	<0.001
Lung cancer	273 (0.65)	358 (0.64)	631 (0.64)	0.96
Osteoporosis	5,620 (13.29)	6,503 (11.68)	12,123 (12.37)	<0.001
Prostate cancer	1,852 (4.38)	2,762 (4.96)	4,614 (4.71)	<0.001

© 2022 Thirukumaran CP et al. *JAMA Health Forum.*

	CJR MSAs	Non-CJR MSAs	Total	p-value[c]
Stroke/ Transient ischemic attack	1,315 (3.11)	1,760 (3.16)	3,075 (3.14)	0.64

Abbreviations: CJR: Comprehensive Care for Joint Replacement Model; MSA: Metropolitan Statistical Area; N: Number; ADRD: Alzheimer Disease and Related Dementias; %: Column percentage; SD: Standard deviation.

Notes: [a] Data from the 2013 Master Beneficiary Summary File – Base Segment; [b] Data from the 2013 Master Beneficiary Summary File – Chronic Conditions Segment. Sum of chronic conditions excluding ADRD; [c] p-values for Kruskal-Wallis tests (for continuous variables) or chi-square tests (for categorical variables) that test for the distribution of characteristics across CJR and non-CJR MSAs; [d] Other: This category is included in Medicare's Master Beneficiary Summary File.

© 2022 Thirukumaran CP et al. *JAMA Health Forum.*

eTable 4. Descriptive statistics for Medicare beneficiaries with or without a diagnosis of Alzheimer Disease and Related Dementias (ADRD) in 2013

	Beneficiaries with ADRD	Beneficiaries without ADRD	Total	p-value[d]
**MSAs: N**	171	171	171	
**Beneficiaries[a]: N**	885,432	3,803,231	4,688,663	
Age in years: Mean (SD)	82.71 (7.70)	75.84 (7.41)	77.14 (7.94)	<0.001
Sex: N(%)				
Female	628,096 (70.94)	2,482,826 (65.28)	3,110,922 (66.35)	<0.001
Male	257,336 (29.06)	1,320,405 (34.72)	1,577,741 (33.65)	
Race/ethnicity: N (%)				<0.001
Asian	30,660 (3.46)	101,163 (2.66)	131,823 (2.81)	
Hispanic	38,179 (4.31)	73,847 (1.94)	112,026 (2.39)	
Non-Hispanic Black	96,232 (10.87)	310,515 (8.16)	406,747 (8.68)	
Non- Hispanic White	703,281 (79.43)	3,225,151 (84.80)	3,928,432 (83.79)	
North American Native	1,859 (0.21)	8,791 (0.23)	10,650 (0.23)	
Unknown	2,924 (0.33)	24,229 (0.64)	27,153 (0.58)	
Other[e]	12,297 (1.39)	59,535 (1.57)	71,832 (1.53)	
Dual-eligible: N (%)	298,740 (33.74)	493,967 (12.99)	792,707 (16.91)	<0.001
Sum of comorbidities[b]: Mean (SD)	5.60 (2.67)	4.09 (2.41)	4.37 (2.53)	<0.001
Hip Replacements[c]: N (%)	3,374 (0.38)	43,776 (1.15)	47,150 (1.01)	<0.001
Knee Replacements[c]: N (%)	6,661 (0.75)	91,304 (2.40)	97,965 (2.09)	<0.001

Abbreviations: CJR: Comprehensive Care for Joint Replacement Model; MSA: Metropolitan Statistical Area; N: Number; ADRD: Alzheimer Disease and Related Dementias; %: Column percentage; SD: Standard deviation; IQR: Inter-quartile range.

Notes: Distribution of comorbidities for Medicare beneficiaries included in this table, and descriptive statistics for hip and knee replacement patients are presented in eTables 1-3. [a] Data from the 2013 Master Beneficiary Summary File – Base Segment and Chronic Conditions Segment; [b] Mean of 23 chronic conditions excluding ADRD obtained from the Master Beneficiary Summary File – Chronic Conditions Segment; [c] Data from the 2013 Medicare Provider Analysis and Review file; [d] p-values for Kruskal-Wallis tests (for continuous variables) or chi-square tests (for categorical variables) that test for the distribution of characteristics across CJR and non-CJR MSAs; [e] Other: This category is included in Medicare's Master Beneficiary Summary File.

© 2022 Thirukumaran CP et al. *JAMA Health Forum.*

eTable 5. Adjusted estimates for parallel trends test from multivariable models examining the use of hip and knee replacement surgeries before the Comprehensive Care for Joint Replacement model was implemented (2013-2015)

	**Hip Replacement**	**Knee Replacement**
	Beta estimate *x 100* [95% CI *x 100*]	Beta estimate *x 100* [95% CI *x 100*]
**Main Effects**		
**Year**	0.03*** [0.02, 0.04]	-0.04*** [-0.05, -0.02]
**MSA Treatment Status**		
Non-CJR MSA	Ref	Ref
CJR MSA	0.36*** [0.19, 0.53]	1.56*** [1.30, 1.83]
**ADRD**		
No	Ref	
Yes	-0.45*** [-0.49, -0.41]	-1.08*** [-1.14, -1.03]
**Two-way interactions**		
Year x MSA Treatment Status	0.02* [0.00, 0.03]	0.02 [0.00, 0.04]
Year x ADRD	-0.04*** [-0.06, -0.02]	-0.02 [-0.04, 0.01]
MSA Treatment Status x ADRD	0.08** [0.03, 0.14]	0.24*** [0.16, 0.31]
**Three-way interactions**		
Year x MSA Treatment Status x ADRD	-0.01 [-0.03, 0.02]	0.00 [-0.03, 0.03]
**N**		
N (patients)	14,227,321	14,227,321
N (MSAs)	171	171

* p<0.05, ** p<0.01, *** p<0.001

Abbreviations: CI: Confidence interval; MSA: Metropolitan Statistical Area; CJR: Comprehensive Care for Joint Replacement model; N: Number; ADRD: Alzheimer Disease and Related Dementias

Notes: Adjusted beta estimates and confidence intervals (multiplied by 100) from patient-level multivariable linear regression models with robust/sandwich estimators of variance. The models controlled for age, sex, race/ethnicity, dual-eligibility, comorbidities, MSA fixed effects, and MSA weights.

© 2022 Thirukumaran CP et al. *JAMA Health Forum.*

eTable 6. Full model estimates from multivariable models examining the use hip or knee replacement surgeries before and after the Comprehensive Care for Joint Replacement model was implemented (2013-2017)

	**Hip Replacements**	**Knee Replacements**
	Beta estimate *x 100* [95% CI *x 100*]	Beta estimate *x 100* [95% CI *x 100*]
**Main Effects**		
**Phase**		
Pre-CJR	Ref	Ref
Post-CJR	0.01 [-0.03, 0.05]	0.19*** [0.16, 0.23]
**MSA Treatment Status**		
Non-CJR MSA	Ref	Ref
CJR MSA	0.43*** [0.28, 0.58]	1.71*** [1.48, 1.93]
**ADRD**		
No	Ref	Ref
Yes	-0.43*** [-0.47, -0.40]	-1.09*** [-1.11, -1.07]
**Two-way interactions**		
Post-CJR x CJR MSA	-0.07** [-0.12, -0.02]	0.02 [-0.01, 0.06]
Post-CJR x ADRD	0.11*** [0.05, 0.17]	0.02 [-0.02, 0.06]
CJR MSA x ADRD	0.08** [0.03, 0.14]	0.24*** [0.21, 0.26]
**Three-way interactions**		
Post-CJR x CJR MSA x ADRD	0.01 [-0.08, 0.09]	-0.03 [-0.09, 0.02]
**Age**	-0.04*** [-0.04, -0.04]	-0.10*** [-0.10, -0.10]
**Sex**		
Male	Ref	Ref
Female	-0.02** [-0.03, -0.01]	0.22*** [0.21, 0.24]
**Race**		
Non-Hispanic White	Ref	Ref
Asian	-0.54*** [-0.55, -0.52]	-0.23*** [-0.26, -0.20]
Hispanic	-0.33*** [-0.34, -0.31]	0.22*** [0.19, 0.26]
Non-Hispanic Black	-0.41*** [-0.42, -0.39]	-0.76*** [-0.78, -0.74]
North American Native	-0.35*** [-0.43, -0.26]	-0.41*** [-0.55, -0.28]
Unknown	0.20*** [0.14, 0.25]	-0.05 [-0.12, 0.02]
Other[b]	-0.51*** [-0.54, -0.49]	-0.21*** [-0.26, -0.16]
**Dual-eligibility**		
No	Ref	Ref
Yes	-0.65*** [-0.66, -0.64]	-1.10*** [-1.11, -1.08]
**Chronic conditions[a]**		
Acute myocardial infarction	-0.37*** [-0.41, -0.33]	-1.04*** [-1.10, -0.99]
Anemia	1.70*** [1.69, 1.71]	2.90*** [2.88, 2.92]
Asthma	0.34*** [0.32, 0.36]	1.08*** [1.05, 1.11]
Atrial fibrillation	0.04*** [0.02, 0.05]	0.13*** [0.11, 0.15]
Benign prostatic hyperplasia	0.47*** [0.45, 0.50]	0.96*** [0.94, 0.99]
Breast cancer	0.26*** [0.23, 0.28]	0.53*** [0.50, 0.57]
Cataract	-0.09*** [-0.10, -0.08]	-0.09*** [-0.11, -0.08]

© 2022 Thirukumaran CP et al. *JAMA Health Forum.*

	Hip Replacements	Knee Replacements
	Beta estimate *x 100* [95% CI *x 100*]	Beta estimate *x 100* [95% CI *x 100*]
Chronic Kidney Disease	-0.36*** [-0.37, -0.35]	-0.63*** [-0.65, -0.61]
Chronic Obstructive Pulmonary Disease	-0.19*** [-0.20, -0.17]	-0.73*** [-0.75, -0.71]
Colorectal cancer	0.04 [-0.01, 0.08]	-0.25*** [-0.30, -0.19]
Congestive Heart Failure	-0.41*** [-0.42, -0.40]	-0.84*** [-0.86, -0.83]
Depression	0.08*** [0.06, 0.09]	0.38*** [0.36, 0.40]
Diabetes	-0.51*** [-0.52, -0.50]	-0.50*** [-0.51, -0.48]
Endometrial cancer	0.68*** [0.59, 0.78]	1.07*** [0.95, 1.20]
Glaucoma	-0.11*** [-0.12, -0.09]	-0.12*** [-0.14, -0.10]
Hyperlipidemia	0.31*** [0.30, 0.32]	0.70*** [0.69, 0.71]
Hypertension	0.45*** [0.43, 0.46]	1.29*** [1.27, 1.30]
Hypothyroidism	0.04*** [0.03, 0.05]	0.24*** [0.23, 0.26]
Ischemic Heart Disease	-0.17*** [-0.18, -0.16]	-0.32*** [-0.33, -0.30]
Lung cancer	-0.34*** [-0.38, -0.30]	-1.11*** [-1.16, -1.06]
Osteoporosis	0.10*** [0.09, 0.12]	-0.01 [-0.03, 0.01]
Prostate cancer	0.31*** [0.28, 0.33]	0.39*** [0.35, 0.42]
Stroke/ Transient ischemic attack	-0.39*** [-0.41, -0.38]	-0.79*** [-0.82, -0.77]
**Year interactions**		
Year	0.03*** [0.02, 0.04]	-0.03*** [-0.04, -0.03]
Year x MSA Treatment Status	0.02* [0.00, 0.03]	NA
Year x ADRD	-0.04*** [-0.06, -0.02]	NA
Year x MSA Treatment Status x ADRD	-0.01 [-0.03, 0.02]	NA
**N**		
N (beneficiary-year)	19,468,093	19,468,093
N (MSAs)	171	171

* p<0.05, ** p<0.01, *** p<0.001

Abbreviations: CI: Confidence interval; MSA: Metropolitan Statistical Area; CJR: Comprehensive Care for Joint Replacement model; N: Number; ADRD: Alzheimer Disease and Related Dementias; NA: Not applicable (because parallel trends assumption not violated)

Notes: Adjusted beta estimates and confidence intervals (multiplied by 100) from patient-level multivariable linear regression models with robust/sandwich estimators of variance. The models controlled for age, sex, race/ethnicity, dual-eligibility, comorbidities, calendar year (and relevant interactions with CJR MSA and ADRD indicator), MSA fixed effects, and MSA weights. The analysis excluded data from 2016 because the CJR was introduced in April 2016, and this implementation precludes the classification of all Medicare beneficiaries into a pre- and post-cohort.
[a] Although the MBSF-CC files report data on 27 chronic conditions, only 23 conditions were included as controls. Alzheimer Disease (included as a part of the ADRD variable [key independent variable]), Hip Fractures (exclusion due to emergent/urgent nature of the condition), and Arthritis (cohort limited to arthritis patients only) were excluded as control variables; [b] Other: This category is included in Medicare's Master Beneficiary Summary File.

© 2022 Thirukumaran CP et al. *JAMA Health Forum.*

eTable 7. Sensitivity Analyses - The Comprehensive Care for Joint Replacement model's association with use of <u>hip and knee replacements</u> for beneficiaries with ADRD compared to beneficiaries without ADRD (2013-2017)

Analysis	Hip replacement		Knee replacement	
	N	*%-point difference*[a] *(95% CI)*	*N*	*%-point difference*[a] *(95% CI)*
Main analysis	19,468,093	0.01 (-0.08, 0.09)	19,468,093	-0.03 (-0.09, 0.02)
Entire cohort	70,956,768	-0.01 (-0.04, 0.02)	70,956,768	-0.02 (-0.06, 0.03)
Intention-to-treat analysis	20,992,092	0.00 (-0.08, 0.08)	20,992,092	-0.02 (-0.07, 0.03)
Medicare's definition of elective surgeries	19,476,933	0.01 (-0.08, 0.09)	19,476,933	-0.03 (-0.15, 0.08)
New ADRD patients	17,102,317	-0.03 (-0.17, 0.11)	17,102,317	-0.09* (-0.18, 0.00)
AD only	19,468,093	0.08 (-0.01, 0.17)	19,468,093	0.04 (-0.09, 0.17)
Logistic regression models	19,468,093	0.03 (-0.08, 0.14)	19,468,093	-0.03 (-0.11, 0.04)
2016 in the post CJR phase	24,598,729	0.00 (-0.07, 0.06)	24,598,729	-0.01 (-0.06, 0.03)

* p<0.05, ** p<0.01, *** p<0.001

Abbreviations: MSA: Metropolitan Statistical Area; CJR: Comprehensive Care for Joint Replacement model; SA: Sensitivity analysis; CI: Confidence interval; N: Number; %: Percentage; ADRD: Alzheimer Disease and Related Dementias; AD: Alzheimer Disease

Notes: Adjusted rates from patient-level multivariable linear regression models with robust/sandwich estimators of variance. The models assessed CJR's association with the use of surgeries for ADRD beneficiaries (versus beneficiaries without ADRD) in CJR MSAs versus non-CJR MSAs. The models controlled for age, sex, race/ethnicity, dual-eligibility, comorbidities, calendar year (and relevant interactions with CJR MSA and ADRD indicator), MSA fixed effects, and MSA weights.

[a] Percentage point difference in the rates of surgeries for beneficiaries with ADRD (versus beneficiaries without ADRD) in CJR MSAs with CJR implementation versus non-CJR MSAs ("triple difference").

© 2022 Thirukumaran CP et al. *JAMA Health Forum.*

eReferences

1. Ryan AM, Kontopantelis E, Linden A, Burgess Jr JF. Now trending: Coping with non-parallel trends in difference-in-differences analysis. *Stat Methods Med Res*. 2019;28(12):3697-3711.

2. Dimick JB, Nicholas LH, Ryan AM, Thumma JR, Birkmeyer JD. Bariatric surgery complications before vs after implementation of a national policy restricting coverage to centers of excellence. *JAMA*. 2013;309(8):792-799.

3. The Lewin Group Inc. CMS Comprehensive Care for Joint Replacement Model: Performance Year 2 Evaluation Report - Appendices. https://downloads.cms.gov/files/cmmi/cjr-secondannrpt-app.pdf. Accessed 09/01, 2020.

© 2022 Thirukumaran CP et al. *JAMA Health Forum.*
